# Bridging the urological divide

**DOI:** 10.1186/1750-9378-6-S2-S4

**Published:** 2011-09-23

**Authors:** Robin Roberts

**Affiliations:** 1 The University of the West Indies, School of Clinical Medicine and Research, The Bahamas Shirley Street, P. O. Box GT-2590, Nassau, Bahamas

## Abstract

The advanced disease clinical presentations, higher morbidity and mortality rates and lack of available treatment options in prostate cancer care, attest to disparities in the delivery and outcomes of urological services in Black men of African lineage in both the Developed and Developing countries. This gap in health care and services in the global management of prostate cancer denotes the urological divide.

Through the experience of a Developing country urologist with a comparative literature review, this presentation defines the determinants of the disparity through deficiencies in human, material and financial resources, as is most prevalent in Developing countries.

Solutions to ending health care disparities must take into account the existing development phase of Third World countries and thus determine whether the Developed countries should export a total service delivery system or seek primarily to advance the competence and skills of the existing Developing country resources.

Collaboration in prostate cancer research has the greatest promise and sustainability of bridging this urological divide and is of mutual benefit to both entities.

## Introduction

The issue of health care disparities is a global phenomenon; it pertains to the gaps in the quality of health and health care across racial, ethnic, sexual orientation and socioeconomic groups [1]. Eliminating these health care gaps or disparities is the foundation of achieving many of the Millennium Development Goals in the Developing world where equitable access and availability of quality healthcare is so evident [2].

A study in the global prostate cancer disparities in Black men underscores the wide disparity in urological health care services between the Developed and Developing countries. Prostate cancer management reviews denote the wide gaps and stark realities of difference in clinical presentations, morbidity, mortality, treatment outcomes and the quality of services available and that can be accessed [3]. The Science of Global Prostate Cancer Disparities in Black seeks to bridge this urological divide [4].

The premise for an understudy of the Developed world as a resource center for bridging this urological divide is centered on the fundamental characteristics of all innovative or revolutionary technological advances that pass the test for sustainability: provide for cost-effective and coordinated care, minimize hospital stay, lend to a minimal access process and to early and quick rehabilitation. It is in this context, this study proposes *that first world medicine was designed for third world countries*[*5*]. Developing countries can least afford the cost of inefficiencies and poor quality care. As bridging the digital divide can advance third world countries through quantum leaps of development, (e.g. cell phone technological applications), *the global collaborations to bridging prostate cancer scientists*, *clinicians and advocates can better understand the etiology of prostate cancer among at-risk Black men*, *and develop effective interventions to address these global urological disparities*[*4*]*.*

This study attempts to highlight the issues and determinants of urological disparities in Developing countries with reference to our English speaking Caribbean experience and Africa.

## Methodology

This presentation was researched from the perspective of providing health services within a Developing country. It’s an overview of the challenges to provide urological care for a small nation state, the Commonwealth of the Bahamas, with a population of over 300,000 people and a transient tourist population in excess of over 4 million per year. Being employed for the past 23 years as the first and solo urologists in the Government’s health care services, providing a standard of care equivalent to that of one’s Developed world’s training is a formidable task.

The literature is reviewed for urologists practicing in Developing countries with similar experiences.

## Results and discussion

The global health disparities are rooted in a lack of available or limited resources: the economics of the relationship between human resources, finances and materials.

### The issue of human resources

Qualified and appropriately trained urologist are limited, scarce and sparsely distributed [6]. The urological manpower need projection for the 21^st^ century indicate clearly the major shortages in Developing countries compared to the Developed ones: 1 to 16,000 in Japan, 1 to 27,000 in the United States, 1 to 250,000 in Great Britain, and 1 to 2.5 million in Ghana. South Africa has 160 for a population of 45 million [7]; Nigeria has an estimated 1 urologist per 3.8 million people [8]. The Bahamas has 2 urological surgeons for a population of over 300,000.

The urologist must function primarily as a generalist. Developing countries will always be at a disadvantage with regards to quality outcomes in comparison to the developing ones with their complement of subspecialists’ services; urological sub-specialization is scarcer still in Developing countries. The urologist is further disadvantaged in not having the disease profile to develop the higher-level competency skills such as performing a radical prostatectomy. There is also a dearth of early prostate cancer suitable for early surgical intervention; in developing countries most urological disease are too advanced and are not amendable for minimal invasive techniques [7][8].

The allied health professional services such as urodynamic nurses and physician assistants and surgical technicians are also scarce and limited. Even having a urologist may be a luxury, in many geographical vicinities the urological service is manned by the general surgeon [9].

The reverse can occur too; in this environment of being a generalist, the urologist may have to undertake the role of being a general surgeon too. There is also the burden of undertaking other professional and administrative responsibilities; manpower resources are scarce universally [8]. The burden of clinical medicine negates also the opportunity to undertake research and to publish; time for CME activities are curbed as well.

Finally there is the issue of reimbursement; government employed physicians in Developing countries are paid very low salaries compared to their counterparts in the Developed world whose salaries are more guaranteed. In the Developing world, the urologist must advance a private practice service and this would normally be the primary income stream. Attention to private practice realities further limit CME and Research activities [8].

The collaborative efforts of the Developed countries provide a tremendous opportunity for the urologist from the Developing world to share and advance his or her skills, knowledge and research to bridge this urological divide. If we are to curb the brain drain of our limited numbers of urologists in the Developing country to the Developed ones, the demand and urgency of initiatives to decrease our urological divide have never been greater.

### The issues of materials

Equipment and supplies are major challenges: What are the most appropriate equipment and materials? Who will maintain them? What is the ease of acquiring replacement parts? And most importantly, who will provide the funding to purchase and maintain them? Equipment needs to be durable, easily maintained and replacement parts readily accessible and available.

In Developing countries, the issue of non-disposable versus disposable materials and supplies requires an evaluation of which one is more cost effective [10]. There is the challenge also in the use of disposable items to have access to replacement materials and supplies. Of equal importance is establishing a reliable supply chain and shipment route.

While it may be ideal to purchase in bulk, there is the issue of costs, warehousing and inventory issues of concern. Disposables for example for endoscopic materials may be managed best by courier services, using a *just in time* process and thus have very little inventory supply.

On the face of it, non-disposable items and re-sterilization may seem the natural philosophy to adopt; the cost of sterilization, durability, and service maintenance may favor disposable products as being more cost-effective [11].

Equipment maintenance is another major challenge in developing countries [12]. Service contracts may be costs prohibitive. Companies may be unreliable in delivering timely services; travel becomes a major factor, some countries may be outside the service routes. Many developing countries lack biomedical technologic support systems and personnel. Preventive maintenance and technical services of medical equipment is a totally new venture and outside the scope of local and national expertise [12].

### The issue of finance

Funding is the most critical factor. Budgets are limited. In most developing countries, the major budgetary expense is personal emoluments. Capital budgets are very limited, if at all. With limited capital for establishing survives, urologist have to be creative to launch services [13].

Establishing new services require capital: equipment and supplies acquisition, transportation, physical plant, human resource requirements, maintenance and ongoing operational costs and inventory management. In the absence of a national health insurance universal access system, urologist desirous of providing state-of-the-art services must undertake creative financing initiatives [14]. Physicians must either contribute funds personally or create Public Private partnerships to provide the capital funding. These private ventures have the disadvantage of limiting universal access; they become costs prohibitive because of the need for user access fees. Developing countries must underscore financial sustainability in the establishing any new service that is particularly technologically driven [15].

### Bridging the urological divide

Ending health care disparities are a herculean task. This issue was addressed comprehensively from the academic perspective several years ago at the Health for All and The Role of Canadian Universities in International Health: Perspectives Issues and problems. The realities are that countries with different economic means and phases of development have different needs [16].

The two scenarios of reducing health care disparities are akin to the famous Confucius quote – “Give a man a fish and feed him for a day; teach him how to fish and feed him for life”

Developing countries where per capital spending on health is a small fraction of the Developed countries GDP require the advancement of an entire health care infrastructure due to their limitations in human and material resources. Missions to Haiti exemplify this and Operations Smile, the worldwide charity for cleft lip repairs in Developing countries [17], [18].

More advancing Developing countries like the English Speaking Caribbean require the acquisition of skills for advancing competency; the requirements are workshops and training programs, fellowships and exchange programs. At the Caribbean Urological Associations professional development programs, advancing skills in percutaneous endoscopic stone surgery was conducted by visiting experts from Britain at the health care facility in Trinidad [19]. Similar training courses were held in urodynamics, incontinence procedures and urological laparoscopic procedures over the ensuing two years; they are ongoing. The advantages were hands on teaching (with minimal issues of patient liability as in North America), minimum incumbencies of travel such as visa requirements and reduced travel expenses. The incentive for urologist to remain in their native country as opposed to migration is invaluable [20]. The exchange and residency programs of the American Urological Association must be recognized as well [21].

The philanthropy in medical equipment and supplies is another avenue for bridging the urological divide. Basic urological equipment and supplies such as resectoscopes and cystoscopies are invaluable gifts. Upgrading and replacement of equipment is slow and philanthropy can be provide invaluable contributions [22]. Many Developing countries are small populations; their purchasing power can be enhanced greatly through relationship with larger countries or health care organizations as well.

### Bridging the urological divide through collaborative research

The discovery of the high prevalence of BR1 and BR2 gene mutations in Bahamian females has great potential with regards to early diagnosis of patients at risk and thus timely interventions, reduced morbidity and mortality [23]. The accessibility and availability of genomic medical services to the Bahamian population through collaborative research provides a quantum leap for the delivery of care to the developing country; it’s a prototype for ending global disparity in breast cancer outcomes.

Through tissue banking and exchange of research collaboration, similar developments could occur for prostate cancer pathologies between the University of Florida, H. Lee Moffitt Cancer Center & Research Institute, Africa and the Caribbean – it’s the essence of The Science of Global Prostate Cancer Disparities in Black Men: "Bridging Gaps Through Research, Education, and Outreach Worldwide".

## Competing interests

The author is a serving Board member of a Public Private Partnership entity contracted to provide private services to the Ministry of Health of the Government of the Bahamas
